# The Accuracy of Cup Placement in Total Hip Arthroplasty (THA) Using an Augmented Reality-Based Navigation System

**DOI:** 10.7759/cureus.59423

**Published:** 2024-04-30

**Authors:** Junya Shimizu, Satoshi Nagoya, Ima Kosukegawa, Arata Kanaizumi, Naoya Nakahashi, Atsushi Teramoto

**Affiliations:** 1 Department of Orthopaedic Surgery, Sapporo Medical University, Sapporo, JPN; 2 Department of Orthopaedic Surgery, Sapporo Kojinkai Memorial Hospital, Sapporo, JPN

**Keywords:** obesity, cup placement accuracy, augmented reality, navigation system, total hip arthroplasty

## Abstract

Background

AR HIP Navigation System^® ^(AR-navi; Zimmer-Biomet, Warsaw, IN) is a portable navigation system employing augmented reality via a smartphone app, which was developed in Japan. We retrospectively analyzed the accuracy of cup placement in total hip arthroplasty (THA) using AR-navi, to investigate whether obesity is associated with an absolute value error in cup placement angle.

Methods

We retrospectively analyzed 45 hips in 43 patients who underwent THA using AR-navi (AR-navi group) and compared them with 45 hips in 45 patients who underwent THA using alignment rods (conventional group).

Results

The mean absolute error of cup placement (AR-navi group vs. conventional group) was found to be 2.60° (±2.11) in radiographic inclination (RI) for the AR-navi group and 4.61° (±3.28) for the conventional group, which indicates significant difference in the AR-navi group compared to that in the conventional group (p = 0.0036). The mean absolute error of radiographic anteversion (RA) was 3.57° (±3.36) for the AR-navi group and 3.87° (±2.97) for the conventional group (p = 0.4732). The mean absolute error of RI was 2.36° (±2.24) in the obese group and 3.16° (±2.03) in the nonobese group, and the mean absolute error of RA was 4.08° (±4.51) and 3.16° (±2.05) in the obese and nonobese groups, respectively.

Conclusions

Cup placement accuracy for THA using AR-navi was 2.60 ± 2.11° for RI and 3.87 ± 2.97° for RA. Compared to THA using the conventional method, the RI installation error was significantly improved with AR Navi. There was no significant difference in the mean absolute error of RI and RA among the obese and nonobese groups.

## Introduction

Failure to achieve accurate cup placement is detrimental to total hip arthroplasty (THA), as it is reportedly associated with increased dislocation rates, limited range of motion, and polyethylene liner wear [1-3]. In 2001, a computed tomography-based (CT-based) navigation system for THA, was approved in Japan. Image-free navigation, which does not require preoperative CT, was developed and applied clinically in 2009. Recently, the trend has been toward portable navigation systems, and the AR HIP Navigation System® (AR-navi; Zimmer-Biomet, Warsaw, IN), a portable system employing augmented reality via a smartphone app, has seen clinical use in THA since 2021. The advantages of AR-navi include its low cost, compact size, and ability to display a radiographic measurement of the cup placement angle based on the functional pelvic plane (FPP). Furthermore, AR-navi allows the patient to be moved to the lateral position for operation after registration in the supine position. To the authors’ knowledge, there has been only one report on navigation errors using AR-navi [4].

Since the AR-navi uses a smartphone camera to register the superior anterior iliac spine on the screen, there is the possibility of error in patients with thick abdominal fat due to the large distance from the skin surface.

The purpose of this study was to evaluate the accuracy of cup placement in THA using AR-navi and to investigate whether obesity is associated with an absolute value error in cup placement angle.

## Materials and methods

Patients

We retrospectively analyzed 45 hips in 43 patients who underwent THA using AR-navi at a single hospital between December 2021 and March 2023 (AR-navi group) and compared them with 45 hips in 45 patients who underwent THA using alignment rods between October 2020 and November 2021 at the same institution as the control group (conventional group). All surgeries were performed using the lateral OCM (Orthopädische Chirurgie München) approach, and all cups were G7OsseoTi® (Zimmer-Biomet).

Patients for subgroup-analysis

Next, we examined whether there was a difference in absolute error between the obese subgroup (BMI ≧ 25) and the non-obese subgroup (BMI < 25) within the AR-navi group.

Radiological measurement

Cup radiographic inclination (RI) and radiographic anteversion (RA) angles were measured with Zed Hip® (Lexi Co., Ltd., Tokyo, Japan) using hip CT conducted one week postoperatively. We calculated the absolute error between cup position angles with postoperative CT and the angle displayed on the AR-navi app. In the conventional group, the target values were 40 degrees for RI and 20 degrees for RA.

Statistical analysis

Fischer's exact test and the Mann-Whitney U test were used for statistical analysis, and a p-value of less than 0.05 was considered significant. All statistical analyses were performed using Prism 10 software (GraphPad Software, Boston, Massachusetts USA, www.graphpad.com).

## Results

The AR-navi group consisted of 45 hips in 43 patients (10 males (23%), 33 females (77%)) with a mean age of 66.7 years (36-93) and a mean body mass index (BMI) of 24.1 kg/m^2^ (16.8-32.0). The causative diseases were osteoarthritis in 32 hips (71%), osteonecrosis of the femoral head in 9 hips (20%), and others in 4 hips (9%). The conventional group consisted of 45 hips in 45 patients (11 males (24%), 34 females (76%)) with a mean age of 62.9 years (46-87) and a mean BMI of 24.6 kg/m^2 ^(17.8-46.2). The causative diseases in the conventional group were osteoarthritis in 33 hips (73%) and osteonecrosis of the femoral head in 12 hips (27%). The AR-navi group was significantly older than the conventional group (p = 0.028) (Table 1).

**Table 1:** Characteristics of the AR-navi group and conventional group BMI: Body mass index, OA: Osteoarthritis, ONFH: osteonecrosis of the femoral head

		AR-navi group	Conventional group	p-value
Number of patients		43	45	ー
Number of hips		45	45	ー
Sex (cases)	Male	10 (23%)	11 (24%)	>0.99
	Female	33 (77%)	34 (76%)	
Mean age yrs. (range)		66.7 (36-93)	62.9 (46-87)	0.028
BMI kg/m^2^ (range)		24.1 (16.8-32.0)	24.6 (17.8-46.2)	0.8205
Cause of surgery (hips)	OA	32 (71%)	33 (73%)	0.1084
	ONFH	9 (20%)	12 (27%)	
	Others	4 (9%)	0 (0%)	

**Figure 1:**
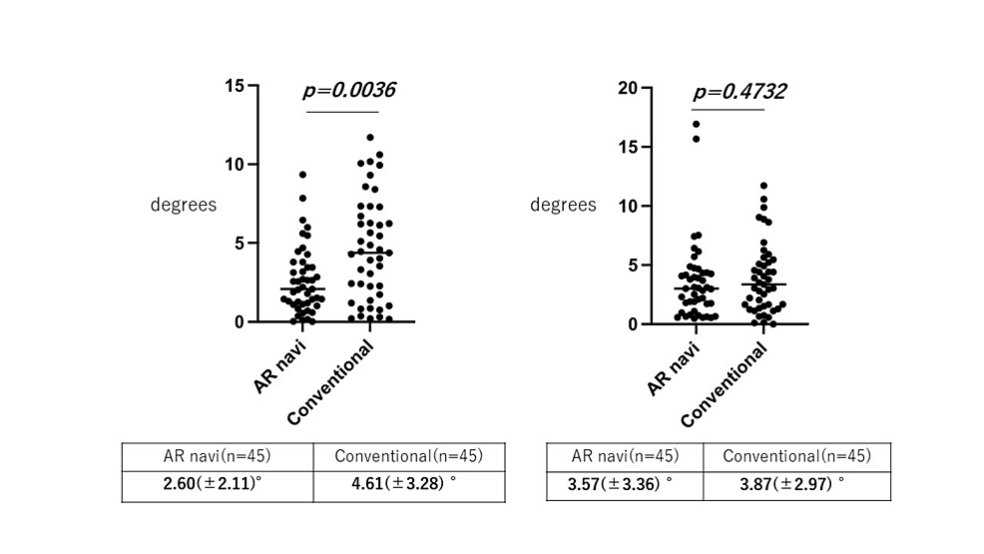
Radiographic inclination and anteversion in the AR-navi group and conventional group Radiographic inclination and anteversion were 2.60° and 3.57°, respectively, in the AR-navi group, and 4.61° and 3.87°, respectively, in the conventional group. Radiographic inclination was significantly smaller in the AR-navi group compared to the conventional group (p = 0.0036).

In the AR-navi group, the average RI and RA values displayed on the screen were 38.4° and 20.8°, respectively. The average RI and RA calculated based on the postoperative CT were 38.6° and 19.1°, respectively. Assessment of the difference in the mean absolute error of cup placement (AR-navi group vs. conventional group) was found to be 2.60° (±2.11) in RI for the AR-navi group and 4.61° (±3.28) for the conventional group. The mean absolute error of RI was significantly lower in the AR-navi group compared to that in the conventional group (p = 0.0036). The mean absolute error of RA was 3.57° (±3.36) for the AR-navi group and 3.87° (±2.97) for the conventional group (p = 0.4732) (Figure 1).

The obese subgroup (BMI ≥ 25) consisted of 20 hips in 20 patients (6 males (30%), 14 females (70%)) with a mean age of 69.4 years (48-85) and a mean BMI of 27.5 kg/m^2^ (25.1-32.0) (Table 2). The nonobese subgroup (BMI < 25) consisted of 25 hips in 23 patients (5 males (20%), 20 females (80%)) with a mean age of 64.2 years (36-79) and a mean BMI of 21.4 kg/m^2^ (16.7-24.7). The mean absolute error of RI was 2.36° (±2.24) in the obese group and 3.16° (±2.03) in the nonobese group, and the mean absolute error of RA was 4.08° (±4.51) and 3.16° (±2.05) in the obese and nonobese groups, respectively (Table 3). None of these differences were significant (RI; p = 0.7995, RA; p = 0.3380).

**Table 2:** Absolute values of navigation error of cup position in the obese and nonobese groups BMI: body mass index

		Obese group (BMI≧25)	Nonobese group (BMI<25)	p-value
Number of cases		20	25	ー
Number of hips		20	25	ー
Sex (cases)	Male	6 (30%)	5 (20%)	0.5001
	Female	14 (70%)	20 (80%)	
Mean age yrs. (range)		69.4 (48-85)	64.2 (36-79)	0.3095
BMI kg/m^2^		27.5 (25.1-32.0)	21.4 (16.7-24.7)	<0.0001

**Table 3:** Absolute values of the navigation error of the cup position in the obese and nonobese groups RI: radiographic inclination, RA: radiographic anteversion

	Obese group (BMI≧25)	Nonobese group (BMI<25)	p-value
RI (degrees±SD)	2.36 (±2.24)	3.16 (±2.03)	0.7995
RA (degrees±SD)	4.08 (±4.51)	3.16 (±2.05)	0.338

## Discussion

AR-navi determines the FPP by reading QR codes attached to pins inserted into the iliac bone to register both super anterior iliac spines, using the camera on a smartphone with a dedicated application. The smartphone is attached to the cup holder and displays the radiographic cup placement angles (RI and RA) based on the FPP. AR-navi is a navigation system developed in Japan and is characterized by its radiographic display, simplicity, and low cost [4].

To the authors’ knowledge, there is only one report on installation errors with AR-navi. Ogawa et al. reported installation accuracies of 1.9° for RI and 2.8° for RA when using AR-navi, both of which were more accurate than the conventional method [4]. Accuracy when using navigation for THA in the lateral decubitus position is shown in Table 4; RI ranges from 1.2 to 3.2° and RA ranges from 1.0 to 3.0° for CT-based methods [5-10]. For image-free navigation, RI ranges from 2.1° to 3.3° and RA ranges from 3.0° to 5.8°, which is less accurate than with CT-based navigation [11-12]. Recently, accelerometer-based navigation has become available, but its accuracy is around 3.2 to 4.1° for RI and 5.9 to 6.8° for RA [10,13-14], indicating that AR-navi tends to be more accurate than other image-free navigation, although not as accurate as CT-based navigation. Considering that AR-navi is compact, inexpensive, and easy to set up, the authors believe that it is a potentially valuable navigation system.

**Table 4:** Accuracy of navigation systems in total hip arthroplasty in the lateral cubitus position

	Authors	Navigation error	
		Radiographic Inclination	Radiographic Anteversion
CT-based navigation	Iwana et al.［5］	1.8±1.6	1.2±1.1
	Yamada et al. ［6］	2.5±2.2	2.3±1.7
	Nakahara et al.［7］	1.2±1.3	1.0±0.8
	Hasegawa et al.［8］	1.9±1.5	3.0±2.3
	Ueoka et al.［9］	1.2±1.0	1.4±1.0
	Tetsunaga et al.［10］	3.2±2.4	3.0±2.3
Image-free navigation	Naito et al.［11］	3.3±2.8	5.8±4.9
	Hasegawa et al.［12］	2.1±1.8	3.0±2.5
Accelerometer-based navigation	Tetsunaga et al.［10］	4.1±3.7	6.8±4.8
	Tanino et al.［13］	3.7±3.3	5.9±3.6
	Tsukamoto et al.［14］	3.2±2.2	6.0±4.1
AR-navi	Ogawa et al.［4］	1.9±1.3	2.8±2.2
	Present study	2.6±2.1	3.6±3.4

In this study, RA was more accurate in the AR-navi group than in the conventional group. In lateral decubitus position THA, the change in pelvic tilt from the supine to the prone position is important for implant placement. Ideally, the pelvic tilt should be 0° relative to the coronal plane in the full lateral position. That is, the operating table should be perpendicular to the line connecting the two teardrops. When an alignment guide is used, an abduction angle of 40° is set as the target value, based on the necessary premise that the pelvic tilt angle relative to the coronal plane is 0°. However, since the pelvis is often tilted cranially or caudally during surgery, installation errors may occur when using an alignment guide. When AR-navi is used, the navigation system corrects for the pelvic tilt angle, so it may allow for more accurate placement.

Some reports have found a negative correlation between obesity and installation accuracy [15-16] while others have found no relationship [11,17-19]. Although this study found no association between obesity and placement error, it is possible that the superior anterior iliac spine and the point registered by the camera may be misaligned in cases with thick abdominal fat when using AR-navi systems that register the superior anterior iliac spine with a smartphone camera, which may be a limitation of AR-navi [17].

Limitations of this study include the lack of information on complications and clinical outcomes, single institution design, and measurements performed by a single examiner.

## Conclusions

We retrospectively assessed the accuracy of cup placement in THA using AR-navi to investigate whether obesity is associated with an absolute value error in cup placement angle. Cup placement accuracy for THA using AR-navi was 2.60 ± 2.11° for RI and 3.87 ± 2.97° for RA. Compared to THA using the conventional method, the RI installation error was significantly improved with AR-navi. There was no significant difference in the mean absolute error of RI and RA among obese and nonobese groups. AR-navi may be a useful tool to improve the accuracy of cup placement with and without obesity.
